# The Use of Sound Recorders to Remotely Measure Grass Intake Behaviour in Horses

**DOI:** 10.3390/ani15152273

**Published:** 2025-08-04

**Authors:** Daisy E. F. Taylor, Bryony E. Lancaster, Andrea D. Ellis

**Affiliations:** 1 Royal (Dick) School of Veterinary Studies, University of Edinburgh, Easter Bush Campus, Roslin, Midlothian EH25 9RG, UK; deftaylor1@gmail.com (D.E.F.T.); bryony.lancaster@ed.ac.uk (B.E.L.); 2 UNEQUI Ltd., Research|Education|Innovation, Launceston, Cornwall PL15 8RT, UK

**Keywords:** horse, foraging, grazing, chew, bite, acoustic recording

## Abstract

Visual observation to record grass intake is time-consuming and labour-intensive. Technological methods, such as activity monitors, have been used but only record head position. This study aimed to evaluate sound recorders attached to headcollars to acoustically measure grass intake behaviour in horses as a low-cost alternative method. Pilot Study 1 assessed 6 × 11 min periods comparing bites/min and chews/min between video footage (VD) and sound recorders (SR). Grazing was identified audibly (SR_ear_) and visually through soundwave pattern software (SR_wav_). Chew rates (SR_ear_: 47 ± 5 chews/min, VD: 43 ± 4 chews/min) were similar between methods. Pilot Study 2 compared hourly grass intake times between SR_wav_ and visual observation (VO) for two horses during a 3 h period. Results showed significant correlation between methods (*rho* = 0.99, *p* < 0.01, Spearman). The main study measured intake behaviour using SR_wav_ and VO methods for three free-ranging horses during 3 h observation periods over multiple days, adding up to 3 × 24 h in winter and in spring (*n* = 48). Mean differences per period between SR_wav_ and VO were 1.8% ± 3 s.d. Foraging duration per period measured with SR_wav_ closely matched VO (*r*^2^ = 0.99, *p* < 0.001). Sound recorders accurately recorded grass intake time and chews in grazing horses during moderate weather conditions.

## Simple Summary

Visually observing horses to record grazing behaviour is time-consuming and labour-intensive. Technology, such as activity monitors, can be an alternative, but they are expensive and less accessible. In this study, the use of sound recorders to measure grazing behaviour in horses was evaluated as a cheaper and less time-consuming alternative. Two pilot studies were conducted to assess how well sound recorders recorded chews/min, bites/min and grass intake durations compared to direct visual observation or video footage. Sound data allowed chews and bites to be measured audibly and visually through soundwave patterns. Results showed good similarity between methods for all measurements, which allowed further application in a large-scale study observing three horses in semi-feral conditions over 24 h for 6 days. Grass intake times measured from sound recorders and visual observations still closely matched. As sound recorders worn by horses accurately measured grazing behaviour in moderate weather conditions without interference with natural behaviour, they are a viable option as an alternative to visual observation.

## 1. Introduction

Horses spend approximately 46–64% of 24 h (approximately 11–15 h) grazing, spread at around 60% during daylight and 40% during darkness, with crepuscular peaks [1,2,3,4,5,6,7]. This diurnal pattern highlights the importance of examining foraging behaviour over 24 h, which is not always feasible. If the ethological requirement to forage is not fulfilled in horses [8], abnormal or stereotypic behaviours (e.g., overeating [9], eating bedding and coprophagy) and digestive tract disorders will occur [10]. Understanding the requirement for food intake behaviour in various situations has therefore become an important part of health research in equids. Studies on equine grazing behaviour have traditionally utilised visual observation to quantify feed intake parameters, such as foraging duration, chew rates and bite rates in field studies and domesticated settings [8]. There are only a limited number of 24 h observations in free ranging horses [1,2,3,4]. Observing long-term grazing behaviour is especially difficult, since it is very time-consuming and horses may be disturbed by observers. Further problems are encountered with limited observers, large herds or poor weather conditions. This likely accounts for the prevalence of studies during daylight hours or summer [5,11,12] and compromises made to observational methods. Most studies employ focal or scan sampling (e.g., one per hour, limited to daylight hours) of the herd or of certain individuals for subsequent extrapolation [13] even though behaviour can vary between horses. These scanning intervals range from 1–30 min but are usually every 5–10 min [5,14,15,16]. Data may be collected through multiple replicates of observation periods over a number of days, which are collated to span a specified time period (e.g., day/night/24 h) [2,5,6]. However, short-term behaviours or onset/termination of long-term behaviours are potentially missed during focal intermittent scanning and observed behaviour may be the latent effects of previous unobserved conditions. Furthermore, restricting data collection to day/night hours may mask equine ultradian rhythms of grazing [17], and it highlights the need for methods allowing continuous observation.

Technology has been employed to remotely monitor foraging behaviour, which allows continuous data collection over longer time periods with increased accuracy and ease compared to visual observation. Telemetry systems (Ethosys) continuously recorded 24 h foraging and activity patterns over an entire year for four Przewalski horses [3]; they have also recently been used on horses at pasture [18]. After use on cattle, the RumiWatch System, which incorporates a noseband pressure sensor to measure chewing cycles, was adapted for equids (EquiWatch System) through integration with headcollars and modification of the behavioural classification algorithm. With this system, chew rates and foraging times were accurately recorded for horses foraging on different feed types and at pasture [19,20].

Acoustic monitoring of foraging behaviour offers another potential method for horses. It has previously been applied to sheep and cattle grazing on grass plots and accurately described foraging times, bite rates and chew rates when compared to synchronised video recordings [21,22]. Even though there is variation in dental morphology between ruminants and equids, grazing produces distinctive sounds. Bite sounds are produced as the incisors grip and rip grass while medio-lateral motion of the temporomandibular joint produces sounds of molar occlusion while chewing [23]. Recently, audio from a grazing horse was used to train a recurrent neural network using a deep learning algorithm to identify bites and chews [24], but it has yet to be applied to further study or long-term observations.

Therefore, the aim of this study was to develop and evaluate the use of simple sound recorders to measure grass intake behaviour in horses at pasture as an alternative to visual observation and other automated continuous activity recorders.

## 2. Materials and Methods

Ethical approval was granted by the Veterinary Research Ethics Committee of the Royal (Dick) Veterinary School, University of Edinburgh (VERC ref: 108.18).

### 2.1. Study Design

**Figure 1:**
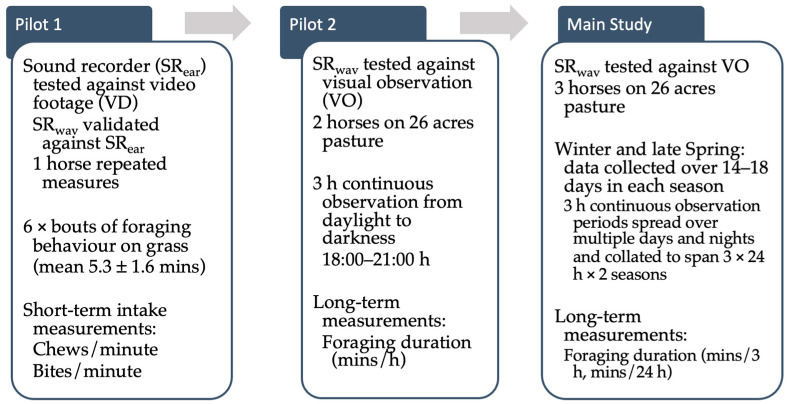
Study design for sound recorder method development and testing for repeatability and reliability (SR_ear_ = behaviour recorded from listening to sound recorders; SR_wav_ = behaviour recorded from adjusted visual soundwaves; h = hour).

Three studies were conducted to develop sound recording methods and to test for repeatability and reliability (Figure 1).

Two pilot studies were conducted in November 2018 to test equipment and compare short-term grass intake measures between sound recorders (SR_ear_: audio data; SR_wav_: visually observing soundwave patterns) and video footage (VD) or visual observations (VO) of grass intake. The aim was to check for initial feasibility of the sound recording output on short-term (Pilot 1: 6 × grazing bouts) and longer-term recordings (Pilot 2: 3 h continuous recording, 2 horses, in light and dark conditions). In Pilot Study 1, video recordings were used to quantify chewing and biting counts while Pilot Study 2 only used visual observations of grass intake behaviour.

The main study was a longitudinal comparative study, observing three Icelandic horses in a small, domesticated herd living outdoors in Shetland, UK in winter and late spring. Three horses (in a herd of eight) wore small sound recorders (SR) to measure the duration of foraging behaviour for comparison with visual observation (VO). The primary researcher was also the sole observer and therefore continuous 12 or 24 h recordings were deemed unreliable and unsafe. Observation periods were limited to fair-weather conditions with windspeeds <6 m/s. Observation periods were continuous 3 h long (period 1: 06:00–09:00 h, 9:00–12:00 h, etc.) with one 24 h period made up from 8 × 3 h recordings collected over 2–3 days, which covered 3 × 24 h periods for winter (December–February) and 3 × 24 h periods in late spring (May–June). Days of prolonged high wind speed or heavy precipitation were omitted.

### 2.2. Animals and Management

For Pilot Study 2 and the main study, two and three adult Icelandic geldings respectively (21 ± 3 years) were used to correlate visual and audio feeding behaviour. They were kept at pasture in Mid Walls, Shetland, UK (60°14′ N and 1°38′ W) during the trails. These horses were part of a mixed-age herd (*n* = 8, 11 ± 9 years) formed in August 2018 consisting of four other geldings and one stallion. The herd was kept outdoors year-round, with the exception of nights with prolonged rain and gale-force winds, when they were occasionally stabled. The horses were normally ridden 3–5 times per week but were not ridden during pilot collection days or throughout the main 6 × 24 h observation periods. During data collection, no additional forage or concentrate was provided.

### 2.3. Study Location

Pilot Study 1 took place in a small grass paddock (0.37 acres) adjoining the main study location, which allowed for visual and camera observations. Pilot Study 2 and the main study occurred on the herd’s usual free-range fields comprised of hill and grassland habitat (26.26 acres). Water was readily available from a running stream and multiple ditches and pools throughout the area. The herd was previously acclimatised to this area and was turned out there for at least a week before data collection commenced.

### 2.4. Measurements and Procedures

#### 2.4.1. Visual Observation and Video Recordings

In Pilot Study 1, a camera (Leica V-Lux 4) was held by the observer in close proximity (<2 m) to the horse’s head and each observation was recorded individually, with videos visually analysed by the observer for counts of chews and bites to quantify chews/min and bites/min. In videos, bites were defined as the visible pulling and ripping of grass with incisors while chews involved visible mediolateral movement of the mandible and activity of the masseter muscle visible above the eye.

For Pilot Study 2 and the main longitudinal study, visual observations recorded the time spent on grass intake behaviour for each horse in the herd (*n* = 8) (without distinction between biting and chewing). Only results for the three horses also wearing sound recorders are included in this report. Night-vision binoculars (Nightfox-100V, Laserware Ltd., Bristol, UK) and a head torch were used for nocturnal observation. Horses could be easily distinguished and were continuously observed for 3 h periods using an ethogram to record behaviours, with the observer talking quietly into a voice recorder (grass intake behaviour only reported in this study) by updating point of time and notable behaviours (e.g., *01:05 h Horse X and Y lift head and stop grazing; 1:16 h X grazing again; 01:18 h Y grazing again*…). Grass intake was defined as grazing with the head lowered with visible chewing and biting movements while standing/walking. During observation, the observer maintained a distance of at least 5 m from horses and walked away if approached.

#### 2.4.2. Sound Recordings

Five minutes before commencing observation periods, sound recorders were set to record, attached to headcollars and fitted to horses. The horses did not react negatively to being approached or having equipment fitted and no food was given to horses at any time. At the end of observation, headcollars were removed.

**Figure 2:**
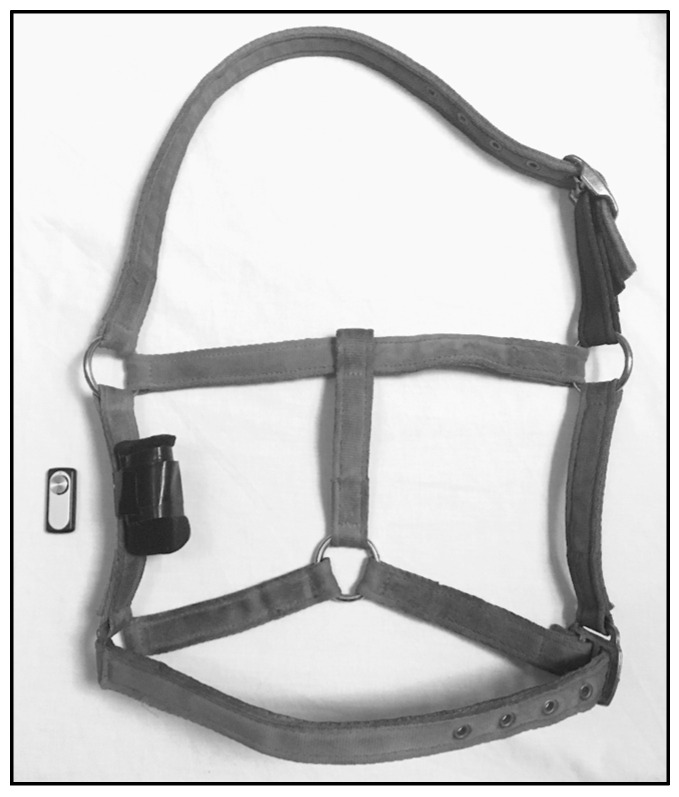
Elements of acoustic recording system with a small sound recorder (60 × 18 × 6 mm, 15 g) incorporated into a field-safe headcollar through placement in a small foam pouch on the inside cheekpiece.

Small 8 GB sound recorders with dimensions of 60 × 18 × 6 mm and a battery capacity of 20 h (Mini voice recorder M1, Milaloko, Shenzhen, China) were used. Recorders were attached to field-safe headcollars (adjustable safety-release padded headcollar, Eclipse, UK). Three mature horses wore recorders, since they were less likely to disturb equipment through play. Recorders were encased in foam microphone covers cut to size (approximately 6 cm) for sound insulation (12 cm shotgun microphone covers, Andoer, Shenzhen, China). These were attached to the inside right cheekpiece of the headcollars using 25 mm duct tape to lay against the horse’s jaw adjacent to the position of the molars (Figure 2). The inside attachment optimised capture of chewing sound and minimized wind interference.

**Figure 3:**
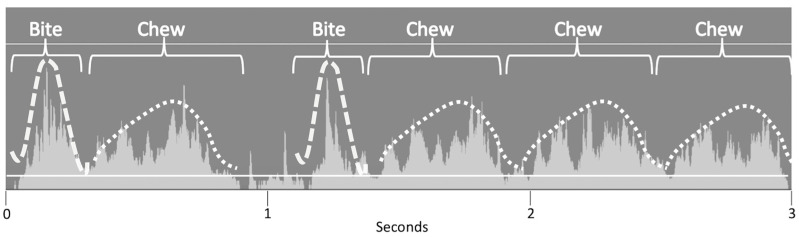
Example of soundwaves (light grey fill) of the stages of mastication, with two different patterns of ingestive behaviour emerging (bite: dashed white line and chew: dotted white line added for illustrative purposes) in a grazing Icelandic horse measured by a sound recorder attached to a headcollar and viewed on editing software with background noise reduced (sound below bottom white solid line).

When grazing, the ripping of grass with incisors during biting and the molar occlusion from mediolateral mandibular movement while chewing produced distinctive audible sounds, but also distinctive visual soundwaves [23]. Editing software (iMovie 10.1.7, Apple, Cupertino, CA, USA) enabled the reduction of background noise and amplification of visual soundwaves of bites and chewing cycles (Figure 3). Bites of grass were identified from soundwave patterns as a higher peak of short duration compared to chews, which produced a longer soundwave at a lower level due to the movement of the mandibular region. In Pilot Study 1, the reliability of utilising these soundwaves to visually quantify chews/min (SR_wav_) was assessed by correlation with chews/min as measured by listening to audio data (SR_ear_). For Pilot Study 2 and the main study, visual soundwaves (SR_wav_) were used to delineate the start and end times of ingestive behaviour and confirmed alongside audible sounds of grass intake (SR_ear_).

### 2.5. Data Analysis

In Pilot Study 1, data derived from video footage, which was used to quantify chews/min and bites/min, were defined as VD (visually observed bite and chew counts from video footage) while auditory data were defined as SR_ear_ (bite and chew counts as heard) and SR_wav_ (chew counts visually quantified from soundwaves—after editing). In Pilot Study 2 and the main study, data derived from visual observation were defined as VO (minutes of observed grass intake) while auditory data were defined as SR_wav_ (minutes of grass intake derived from visual identification of soundwave patterns with confirmation from audible sounds of grass intake as heard).

Statistical software SPSS Version 22 (IBM, Armonk, NY, USA) and JASP Version 0.9.2 (University of Amsterdam, Amsterdam, the Netherlands) were used for data analysis. Significance was set to *p* < 0.05. Datasets were tested for normality with a Shapiro–Wilk test. Descriptive statistics presented results using means and standard errors (unless otherwise stated) for variables according to recording method or season where applicable.

In Pilot Study 1, data were analysed as not normally distributed due to the small sample size. From the first pilot study, chew rates established from audio data (SR_ear_) were correlated with chew rates established from visually observed soundwaves (SR_wav_) with a Spearman rank correlation to assess the reliability of utilising soundwaves to quantify audio data for long-term measurements. A Wilcoxon signed-rank test compared differences of audio recordings (SR_ear_) with visual observations (VO) for chews/min and bites/min. For the second pilot study, the grass intake durations (min per h) of two horses measured from audio recordings (SR_wav_) were correlated with those visually observed (VO) with a Spearman rank correlation.

For the main study, all data were normally distributed and grass intake durations (min per 3 h period) of the three horses measured from audio recordings (SR_wav_) were correlated against their visually observed grass intake times (VO) using a Pearson correlation. This agreement was further evaluated through a Bland–Altman plot: differences between visual and audible data were plotted against the means of both methods. The bias (mean difference) and limits of agreement (95% confidence intervals) were also plotted, and a significant bias was determined if the line of equality did not fall within the limits of agreement (95% confidence intervals [CI]).

## 3. Results

### 3.1. Pilot Method Development

**Figure 4:**
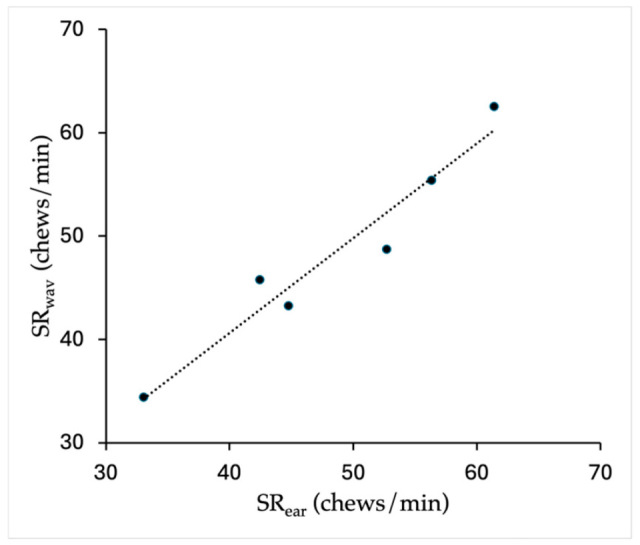
Pilot Study 1: Chews/min as measured from visual counts of chews from soundwaves (SR_wav_) or counts of chews heard (SR_ear_) from one grazing horse wearing a sound recorder for six grazing bouts (5.3 ± 1.6 min) (linear relationship line shown).

There was a strong correlation between SR_ear_ and SR_wav_ in Pilot Study 1 (*p* = 0.017, *n* = 6, %cv = 46%, Spearman Rank, *rho* = 0.943; Figure 4), which enabled the use of SR_wav_ for quantifying grass intake times in Pilot Study 2 and the main study, where only SR_wav_ was used for sound recorder data with some SR_ear_ confirmation at the beginning and end of feeding bouts or when unusual soundwaves were seen.

**Figure 5:**
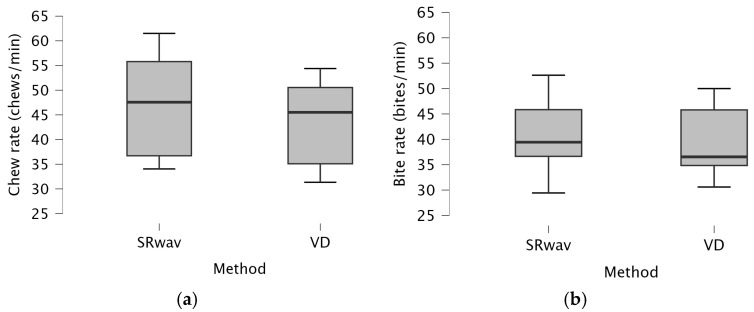
Pilot Study 1: Short-term intake rates of an Icelandic horse measured by visual observation (VD) and audio data from sound recorders (SR_ear_) during six short grazing bouts (5.3 ± 1.6 min) for (**a**) chews/minute; (**b**) bites/minute.

During short-term grazing bouts, chewing rates showed a slight difference between video (VD; 43 ± 4 chews/min) and audio recordings (SR_ear_; 47 ± 5 chews/min), as shown in Figure 5a (Wilcoxon, *W*(5) = 0.0, *p* = 0.03). However, a Wilcoxon test does not take account of ‘magnitude’ of differences and Figure 5a highlights that this is likely a coincidental statistical difference in this small sample size. Bite rates were not different between video (VD; 39 ± 3 bites/min) and audio recordings (SR_ear_; 41 ± 3 bites/min), as shown in Figure 5b (*W*(5) = 3, *p* = 0.1596).

**Figure 6:**
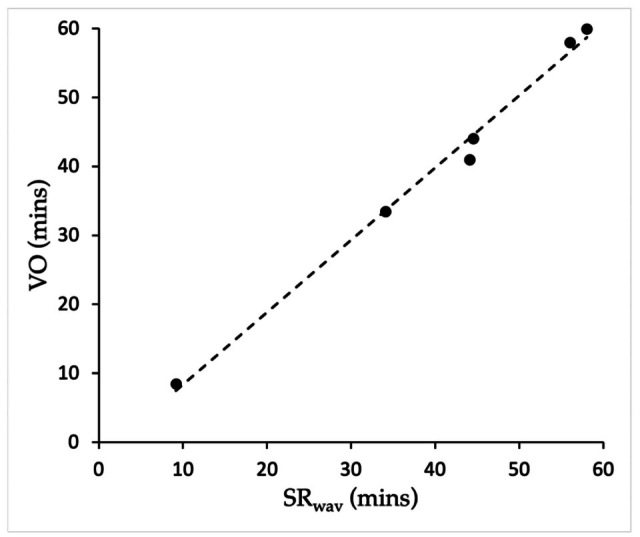
Pilot Study 2: Hourly grass intake times (min per hour) from 18:00–21:00 h of two Icelandic horses at pasture measured from sound recorders (SR_wav_) and visual observation (VO) (linear relationship line shown).

In Pilot Study 2 (3 × 1 h observations), visually observed (VO) hourly grass intake times (min/h) closely matched times measured from sound recordings (SR_wav_), as shown in Figure 6 (Spearman Rank; *rho* = 0.999, *p* = 0.003).

### 3.2. Grass Intake Patterns According to Recording Method

There were no significant differences between any of the SR and VD/VO recordings apart from a slight difference in Pilot 1 on a non-parametric test. Summary data of all three studies can be seen for comparison in Table 1.

**Figure 7:**
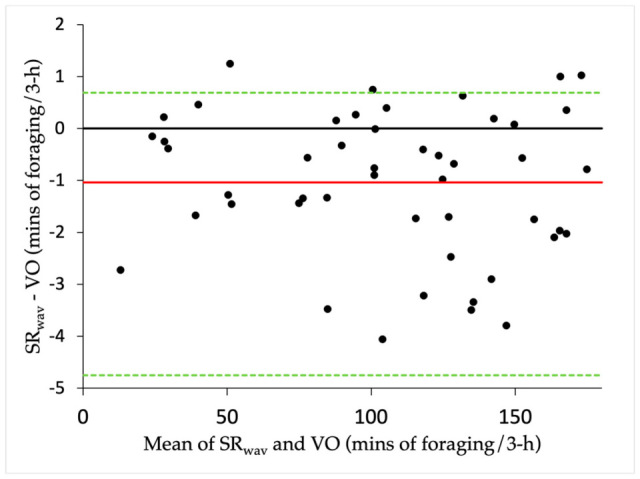
Agreement of measurements from sound recordings (SR_wav_) and visual observation (VO) for foraging duration (minutes) over 3 h observation periods of three horses grazing at pasture. A Bland–Altman plot shows agreement between the two methods (solid red line denotes mean difference; solid black line denotes the line of equality; dotted green lines denote the 95% limits of agreement).

Correlations from the main study show that the mean grass intake times of each horse per period measured from sound recordings closely matched visual observations (Pearson correlation; *r*^2^ = 0.999, *n* = 48, *p* < 0.001). The comparison between sound recordings and visual observation was analysed further with a Bland–Altman plot (Figure 7).

A bias of −1.01 min was shown with sound recorders measuring slightly shorter foraging durations than visual observations during 3 h observation periods. The upper and lower limits of agreement were 0.69 and −4.75 min, respectively. This bias was not considered significant, since the line of equality was within the 95% confidence interval.

## 4. Discussion

This study aimed to develop and evaluate the use of sound recorders to measure grass intake behaviour. This was incorporated into a study assessing the seasonal variation of 24 h time budgets of Icelandic horses at pasture in winter and spring. In short-term trials, sound recorders recorded short-term intake rates nearly identical to those visually observed. During long-term observation over 24 h, foraging durations recorded by sound recorders were close to visual observations and afforded increased accuracy for any foraging/chewing behaviour missed visually when using ‘head down’ as an indicator. An average of 1 min/3 h period of foraging recorded visually was not ‘heard’ as chewing or biting behaviour from the sound recorder transcription (range from 4.1 min to −1.2 min/period, *n* = 48). This is most likely because the observer could not always confirm grass intake behaviour from a distance, especially at night while observing multiple horses with heads down.

### 4.1. Short-Term Intake Rates

The purpose of Pilot Study 1 was to explore the feasibility of using audio recorders instead of visual observations in order to move on to more long-term observations. Chewing rates measured from the sound recorder were nearly equivalent to visual observations from video recordings. Visual observation may be difficult on certain native breeds of equines with thicker coats and long manes which cover the mandibular area. However, recording audio from a position close to the mouth detected all chewing and biting motions even if another horse was grazing in close proximity.

In this study, chewing rates of 47 ± 11 (s.d.) chews/minutes were shown. Chewing rates of 60–80 chews/min have been shown in previous research on horses fed conserved forages and concentrates based on visual observation [8] and the EquiWatch System [19], which utilises a noseband pressure sensor to detect chewing. In grazing horses, studies have shown comparable chewing rates of 56–88 chews/min and bite rates of 9–57 bites/min [20,25]. However, ‘chewing rates’ as measured by the EquiWatch System on grazing horses [20] included prehensile bites in addition to chews in their measurements. Developing the visual soundwave system to apply to different breeds and feed types could be a future application.

The additional easy distinction between soundwaves for bites and chews highlights that while grazing horses take in forage continuously [8], they seem to often interrupt the chewing cycle to take a bite from the grass sward. In this area, further research is required to fully validate sound recorders for measuring short-term intake parameters in various types of feedstuffs. For the purpose of assessing grass intake parameters for free-ranging horses, the two pilot studies were highly accurate, which allowed us to move onto the full main study. Sound recorders, therefore, offer a promising alternative to visual observations in domesticated horses.

Similarly to sound recorders, the EquiWatch System is incorporated with a headcollar, which allows adjustment to individual horses. Foraging activity is detected through a pressure sensor and triaxial accelerometer built into the noseband [19,20]. This design is bulkier compared to the sound recorders used here, which may make it difficult to fit to smaller equid breeds. The EquiWatch System showed high agreements with visual observations for grazing times (concordance correlation coefficient = 0.997) and total chew counts (CCC = 0.979). However, it cannot fully distinguish between chews and bites, since the system was initially developed for cattle (RumiWatch) [20].

Pilot Study 1 highlighted that sound recorders can differentiate between bite and chewing sounds but either require someone manually listening to recordings or observing/counting all soundwaves visually unless a program algorithm could be developed for differentiation in the future. Acoustic assessment of ingestive behaviour has successfully predicted dry matter (DM) intakes in cattle and sheep grazing on micro-sward plots. This method also allowed differentiation between chews and bites and the identification of chew-bites (a simultaneous bite and chew during a jaw movement cycle). Chewing sound energy showed a linear relationship to DM intake in cattle and sheep across different sward types offered in trays [21,22], but has not yet been assessed at pasture. However, grass was presented in plastic pots rather than grazing from the ground. Galli et al. [21] quantified chewing sound energy and developed calculations to determine predictors of DM intake. A similar methodology could be applied to equids using sound recorders.

### 4.2. Grass Intake Behavioural Patterns

The sound recorders accurately recorded grass intake durations during 3 h observation periods over all times of day across multiple seasons. The main study showed a correlation coefficient of 0.99 between visual observations and sound recorder data for grass intake times. The EquiWatch System uses pressure sensors to record mandibular movement, but was not integrated with field-safe headcollars [20]. The EquiWatch System recorded a correlation coefficient of 0.997 between its sensor and visual observations in chewing times [20]. However, the small size of recorders used in this study minimised disturbance to natural behaviour, and their incorporation with field-safe headcollars with a Velcro break-away strap reduced safety risks. Though sound recorders encased in microphone covers were placed against the horse’s cheek, no signs of rubbing or lesions were observed throughout the study, and the headcollar was fitted to allow unrestricted movement of the jaw. However, for longer observation periods, additional measures may have to be considered to ensure comfort for the horse. Inside placement of microphones eliminated some wind interference. Battery life of the small recorders used in this study was 20 h. Therefore, sound recorders could be utilised as a cheaper alternative, with only a small increase in size for battery capacity.

The ETHOSYS telemetry system incorporates two sensors to detect acceleration and head position into a collar worn around the horse’s neck, but showed a correlation between visual observations (84.6 ± 14.7%) for feeding [26], which is lower than the correlation coefficient shown in this study (99%). The ETHOSYS system defined feeding activity as specific head movements while lowered (interpreted as chewing) during standing or walking. However, this may overestimate grazing behaviour if horses lowered their head without grazing, or underestimate it if chews were too infrequent or the horse was chewing while its head was raised [26]. ETHOSYS weighs 300 g (weight of a small mobile phone) excluding the collar [3], which could become caught on something and cause injury to the horse. The sound recorders used in this study weighed only 15 g.

More recent methods for indirect observation for foraging, such as EquiWatch [20], video cameras [24] and sound recorders are likely more accurate as they are in better positions to detect intake parameters than the ETHOSYS neck collar. However, ETHOSYS allows continuous detection of overall activities (standing, walking, feeding, lying) for long periods of time, allowing identification and comparison of behavioural patterns [3,26]. For foraging behaviour only, sound recorders are much cheaper and are easy to attach. Data were retrievable in simple formats without the requirement of an app and could be analysed with user-friendly audio software. Editing improved the clarity of foraging sounds to allow both visual and audible identification of foraging behaviour.

Data collection was limited to calm weather due to the effect of wind on audio recordings despite testing several designs for sound insulation. The final design utilised shotgun microphone covers, which reduced most wind sounds on audio files. However, windspeeds of approximately >7 m/s made it almost impossible to distinguish chewing sounds. Recorders remained dry even during light showers, since data were mostly collected during fair weather, but would need to be fully wind- and waterproof to extend data collection to all weather conditions. Even though sound recorders were operating continuously during observation, certain non-foraging behaviours, such as resting, playing and walking, could be distinguished, but not with the same clarity as foraging. This limits its current use to solely monitoring foraging/food intake behaviour, unlike ETHOSYS [3] and triaxial accelerometers, which can identify locomotion, standing and lying [27].

### 4.3. Future Applications of the Method

Currently, the use of sound recorders would likely be limited to domesticated horses habituated to wearing headcollars, since they require removal for data retrieval and charging. Some visual observation may be required, since juvenile horses attempted to dislodge headcollars and sound recorders during play, which required brief interference from the observer. However, this may not be an issue with a mature herd or horses in separate enclosures, though recorders may be dislodged during head-rubbing behaviours. Therefore, regular visual checks should be made to ensure the safety of horses and functioning of equipment.

Improving the understanding of the 24 h pattern of intake behaviour in domesticated horses can enable the development of more species-specific feeding systems, especially when they are stabled and used for exercise. In addition, for domesticated horses there is often a need to uphold good welfare by facilitating foraging behaviour while reducing caloric intake due to obesity. The development of slow feeders [28,29] and behaviour observation apps [30] highlights the demand for owners to be able to record daily feeding patterns in an applied situation. A simple sound recorder that eventually could be linked to an app transferring feeding to a visual output will allow owners to notice sudden changes and may give advance warning of digestive or dental problems.

One study also used audio data from a grazing horse and developed a deep learning algorithm (Recurrent Neural Network) to identify and detect chews and bites with an accuracy of 93.2% and 87.8%, respectively [24], but has yet to be used to measure feed intake parameters. These areas of research show an encouraging direction for the future use of acoustic monitoring in behavioural studies. The use of audio-visual analysis of soundwaves in our study was an unexpected addition and was very much at an initial ‘look-see’ stage, but soundwaves have been explored in much more depth in other species, primarily for vocalisation analysis [31]. These techniques could be applied much more to analyse ingestive sound patterns of horses to gain accurate distinctions between chews, bites and ‘chew-bites’ (which can occur simultaneously) for a variety of feedstuffs, breed or size of horses and management systems.

## 5. Conclusions

Acoustic analysis of foraging through sound recorders showed high levels of agreement with visual observations over 24 h between multiple seasons and periods varying in grazing intensity. Small sound recorders attached to headcollars could offer a low-cost and non-invasive method of measuring foraging behaviour in pasture and stabled settings. Their use could also be expanded to quantify bite and chew behaviour patterns according to feedstuffs and feed presentation across different breeds. Furthermore, sound recorders could be used to assess welfare conditions through determining whether foraging times meet horses’ ethological requirements or evaluating the presence of oral stereotypies or dental abnormalities.

## Figures and Tables

**Table 1:** Grass intake data from sound recorders (SR) and video (VD) or visual (VO) observations for Pilot Study 1 (chews/min and bites/min) and grass intake times (min) from Pilot Study 2 (6 × 1 h observations, two horses) and the main study (48 × 3 h observations, 6 × 24 h observations, three horses).

	Variable	Method	*n*	Mean	SD	Min.	Max.
Pilot Study 1	Chews/min	SR_ear_	6	47.1	11.6	34.1	61.5
		VD	6	43.4	9.7	31.3	54.4
	Bites/min	SR_ear_	6	40.8	8.3	29.4	52.6
		VD	6	39.5	8	30.6	50
Pilot Study 2	Grass intake per 1 h (min)	SR_wav_	6	41	17.9	9.2	58.1
		VO	6	40.8	18.8	8.5	60
Main Study	Grass intake per 3 h period (min)	SR_wav_	48	105.5	45.7	11.5	174.4
		VO	48	106.8	46	14.2	175.1
	Grass intake per 24 h total (min)	SR_wav_	6	843.7	161.3	585.6	994.1
		VO	6	854.1	163.7	594.5	1014.9

Values shown represent the number of replicates (*n*), the mean, standard deviation (SD), minimum and maximum.

## Data Availability

The data presented in this manuscript are available upon request from the corresponding author.

## References

[1] Duncan P. (1985). Time-Budgets of Camargue Horses Iii. Environmental Influences. Behaviour.

[2] Boyd L.E., Carbonaro D.A., Houpt K.A. (1988). The 24-Hour Time Budget of Przewalski Horses. Appl. Anim. Behav. Sci..

[3] Berger A., Scheibe K.-M.M., Eichhorn K., Scheibe A., Streich J. (1999). Diurnal and Ultradian Rhythms of Behaviour in a Mare Group of Przewalski Horse (Equus Ferus Przewalskii), Measured through One Year under Semi-Reserve Conditions. Applied Animal Behav. Sci..

[4] Vulink T.J. (2001). Hindgut Fermentation Is Not an Evolutionary Dead End: Comparative Feeding Ecology of Cattle and Horses. Hungry Herds: Management of Temperate Lowland Wetlands by Grazing.

[5] Souris A.C., Kaczensky P., Julliard R., Walzer C. (2007). Time Budget-, Behavioral Synchrony- and Body Score Development of a Newly Released Przewalski’s Horse Group Equus Ferus Przewalskii, in the Great Gobi B Strictly Protected Area in SW Mongolia. Appl. Anim. Behav. Sci..

[6] Edouard N., Raldine Fleurance G., Dumont B., Baumont R., Duncan P., Edouard N., Fleurance G., Dumont B., Baumont R., Duncan P. (2009). Does Sward Height Affect Feeding Patch Choice and Voluntary Intake in Horses?. Appl. Anim. Behav. Sci..

[7] Mayes E., Duncan P. (1986). Temporal Patterns of Feeding Behaviour in Free- Ranging Horses. Behaviour.

[8] Ellis A.D., Ellis A.D., Longland A.C., Coenen M., Miraglia N. (2010). Biological Basis of Behaviour and Feed Intake. The Impact of Nutrition on the Health and Welfare of Horses. EAAP Publication No 128.

[9] Argo C.M., Cox J.E., Lockyer C., Fuller Z. (2002). Adaptive Changes in the Appetite, Growth and Feeding Behaviour of Pony Mares Offered Ad Libitum Access to a Complete Diet in Either a Pelleted or Chaff-Based Form. Anim. Sci..

[10] Galinelli N., Wambacq W., Broeckx B.J.G., Hesta M. (2021). High Intake of Sugars and Starch, Low Number of Meals and Low Roughage Intake Are Associated with Equine Gastric Ulcer Syndrome in a Belgian Cohort. Anim. Physiol. Nutr..

[11] Magnússon J., Thorhallsdóttir A.G. (1994). Horse Grazing in Northern Iceland–Behavior and Habitat Selection. Livest. Prod. Sci..

[12] Boyd L., Bandi N. (2002). Reintroduction of Takhi, Equus Ferus Przewalskii, to Hustai National Park, Mongolia: Time Budget and Synchrony of Activity Pre-and Post-Release. Appl. Anim. Behav. Sci..

[13] Altmann J. (1974). Observational Study of Behavior: Sampling Methods. Behaviour.

[14] Keiper R.R., Keenan M.A. (1980). Nocturnal Activity Patterns of Feral Ponies. J. Mammology.

[15] Houpt K.A., O’Connell M.F., Houpt T.F., Carbonaro D.A. (1986). Night-Time Behaviour of Stabled and Pastured Peri-Parturient Ponies.Pdf. Appl. Anim. Behav. Sci..

[16] Van Dierendonck M.C., Bandi N., Batdorj D., Dügerlham S., Munkhtsog B. (1996). Behavioural Observations of Reintroduced Takhi or Przewalski Horses (Equus Ferus Przewalskii) in Mongolia. Appl. Anim. Behav. Sci..

[17] Martin A.M., Elliott J.A., Duffy P., Blake C.M., Attia S.B., Katz L.M., Browne J.A., Gath V., McGivney B.A., Hill E.W. (2010). Circadian Regulation of Locomotor Activity and Skeletal Muscle Gene Expression in the Horse. J. Appl. Physiol..

[18] Collas C., Dumont B., Delagarde R., Martin-Rosset W., Fleurance G. (2015). Energy Supplementation and Herbage Allowance Effects on Daily Intake in Lactating Mares. J. Anim. Sci..

[19] Werner J., Umstatter C., Zehner N., Niederhauser J.J., Schick M. (2016). Validation of a Sensor-Based Automatic Measurement System for Monitoring Chewing Activity in Horses. Livest. Sci..

[20] Weinert J.R., Werner J., Williams C.A. (2020). Validation and Implementation of an Automated Chew Sensor–Based Remote Monitoring Device as Tool for Equine Grazing Research. J. Equine Vet. Sci..

[21] Galli J.R., Cangiano C.A., Milone D.H., Laca E.A. (2011). Acoustic Monitoring of Short-Term Ingestive Behaviour 1 and Intake in Grazing Sheep. Livest. Sci..

[22] Galli J.R., Cangiano C.A., Pece M.A., Larripa M.J., Milone D.H., Utsumi S.A., Laca E.A. (2018). Monitoring and Assessment of Ingestive Chewing Sounds for Prediction of Herbage Intake Rate in Grazing Cattle. Animal.

[23] Bonin S.J., Clayton H.M., Lanovaz J.L., Johnston T. (2007). Comparison of Mandibular Motion in Horses Chewing Hay and Pellets. Equine Vet. J..

[24] Nunes L., Ampatzidis Y., Costa L., Wallau M. (2019). Horse Foraging Behavior Detection Using Recurrent Neural Networks. https://isaim2020.cs.ou.edu/papers/ISAIM2020_Agriculture_Nunes_etal.pdf.

[25] Edouard N., Duncan P., Dumont B., Baumont R., Fleurance G. (2010). Foraging in a Heterogeneous Environment-An Experimental Study of the Trade-off between Intake Rate and Diet Quality. Appl. Anim. Behav. Sci..

[26] Scheibe K.M., Schleusner T., Berger A., Eichhorn K., Langbein J., Dal Zotto L., Streich W.J. (1998). ETHOSYS (R)—New System for Recording and Analysis of Behaviour of Free-Ranging Domestic Animals and Wildlife. Appl. Anim. Behav. Sci..

[27] Maisonpierre I.N., Sutton M.A., Harris P., Menzies-Gow N., Weller R., Pfau T. (2019). Accelerometer Activity Tracking in Horses and the Effect of Pasture Management on Time Budget. Equine Vet. J..

[28] Hart R., Bailey A., Farmer J., Duberstein K. (2024). Chewing Analysis of Horses Consuming Bermudagrass Hay in Different Styles of Slow Feeders as Compared to Loose Hay. J. Equine Vet. Sci..

[29] Roig-Pons M., Bachmann I., Briefer Freymond S. (2025). Slow-Feeding Dispensers for Horses: Who, How and Why?. J. Vet. Behav..

[30] Eerdekens A., Papas M., Damiaans B., Martens L., Govaere J., Joseph W., Deruyck M. (2024). Automatic Early Detection of Induced Colic in Horses Using Accelerometer Devices. Equine Vet. J..

[31] Fitch W.T., Anikin A., Pisanski K., Valente D., Reby D. (2025). Formant Analysis of Vertebrate Vocalizations: Achievements, Pitfalls, and Promises. BMC Biol..

